# Psychological Factors as Predictors of Suicidal Ideation among Adolescents in Malaysia

**DOI:** 10.1371/journal.pone.0110670

**Published:** 2014-10-23

**Authors:** Norhayati Ibrahim, Noh Amit, Melia Wong Yuin Suen

**Affiliations:** Faculty of Health Sciences, National University of Malaysia (UKM), Kuala Lumpur, Malaysia; Catholic University of Sacred Heart of Rome, Italy

## Abstract

**Background:**

There has been a drastic increase in the rate of suicides over the past 45 years in Malaysia. The statistics show that adolescents aged between 16 and 19 years old are at high risk of committing suicide. This could be attributed to issues relating to the developmental stage of adolescents. During this stage, adolescents face challenges and are exposed to various stressful experiences and risk factors relating to suicide.

**Method:**

The present study examined psychological factors (i.e., depression, anxiety and stress) as predictors for suicidal ideation among adolescents. A cross-sectional study was conducted on 190 students (103 males and 87 females), aged 15 to 19 years old from two different schools in Kuala Lumpur. The Depression Anxiety Stress Scale 21-item version (DASS-21) was used to measure depression, anxiety and stress among the students, and the Beck Scale for Suicide Ideation (BSS) to measure suicidal ideation. The data were analysed using Pearson's correlation and multiple regression analysis.

**Results:**

The results show that 11.10%, 10.00%, and 9.50% of the students reported that they were experiencing severe depression, anxiety and stress, respectively. There were significant correlations between depression, anxiety, and stress with suicidal ideation. However, only depression was identified as a predictor for suicidal ideation.

**Conclusion:**

Hence, this study extends the role of depression in predicting suicidal ideation among adolescents in the Malaysian context. The findings imply that teenagers should be assisted in strengthening their positive coping strategies in managing distress to reduce depression and suicidal ideation.

## Introduction

Suicide can be considered as a ‘silent enemy’. Although it is common to find that people do not admit to having suicidal thoughts, some of them have the potential to die by committing suicide. In fact, there is a common public misperception that only those who are diagnosed with depression are at risk of committing suicide. However, almost 40% of the people who commit complete suicide are not clinically depressed, which indicates that various psychological conditions could potentially increase the risk of suicide. These include post-traumatic stress disorder, eating disorders and bipolar disorder [1]. In 2010, suicide was found to be the tenth leading cause of death (38,364 people a year) in the United States of America [2]. In fact, 90% of the suicides are committed by people suffering from psychological disorders, 60% are associated with mood disorders, 25%–50%% are associated with alcohol use and abuse, and 10% are associated with borderline personality disorder [3].

In developing countries, there is a dearth of research on suicide and suicidal ideation. This could be attributed to the taboo related to suicide. For instance, in Malaysia, committing suicide is a taboo and the topic is not openly discussed. In recent years, the suicide rate has increased and appears to be an emerging social problem in Malaysia. Indeed, statistics show that the suicidal rate has increased by 60% over the past 45 years and it is estimated that 140 people attempt suicide daily [4]. The Malaysian Psychiatric Association estimated that seven people, who mostly comprise youth and young adults, are killing themselves daily [5]. This is indeed a shocking statistic. It implies that within 24 hours, seven lives are lost due to suicide.

According to the National Health and Morbidity Survey conducted by the Ministry of Health Malaysia [6], 6.3% of the participants reported having suicide ideation. In this survey, it was found that the youngest aged group – 16 to 19 years old – was most disposed to suicidal thoughts followed by the 20 to 24 years age group. The findings also showed that females reported higher suicidal ideation. However, there was no difference between the urban and rural population concerning suicidal ideation [5], [6]. In addition, the National Suicide Registry Malaysia (NSRM) estimated that between January and August 2010, a total of 425 people had committed suicide [7]. In addition, a local study by Tam et al. [8] reported that there were 445 suicide cases in Malaysia for the first eight months of 2010 compared to 290 cases in 2008. These figures were only based on legal cases or cases reported to the police, indicating that there are still many unreported cases. Therefore, it is important to examine the nature and extent of suicidal ideation in the Malaysian context.

There are various risk and protective factors associated with suicide and suicidal ideation among young people [9]. For instance, Gutierrez and Osman [9] classified the risk factors into individual risk factors and family risk factors, and protective factors into personal protective factors and environmental protective factors. However, notwithstanding the above factors, the psychological related factors play an important role in understanding the psychological wellbeing of those who have the potential to be involved in suicide and suicidal ideation; and, understanding these factors, helps to determine the tendency of young people to engage in suicidal ideation and suicide-related behaviour [10].

Some of the major psychological factors associated with suicidal ideation are depression, anxiety, and stress. Most of the findings on the relationship between psychological factors and suicide as well as suicidal ideation are based on the evidence of the psychological autopsy conducted among those who have committed suicide [11]. Depression is one of the important psychological factors related to suicide and suicidal ideation. Evidence from the psychological autopsy showed that, at the time of suicide or attempted suicide, some people were suffering from depression [11]. Furthermore, research conducted in a clinical setting, which was based on clinical screening of approximately 1000 patients in a cardiology clinic for depression and suicidality, showed that 109 patients (12%) expressed suicidal ideation [12]. Research also indicated that depression is positively associated with suicidal ideation [13], [14], [15]. The depression could be attributed to individuals' feelings of hopelessness, helplessness, and a lack of social support and coping skills when they face difficulties and stressful life experiences.

Another common risk factor for suicidal ideation is anxiety. Anxiety is one of the most common psychiatric illnesses seen in general practice [16]. Although anxiety is found to be positively associated with suicidal ideation, the relationship between anxiety and suicidal ideation is inconclusive [17]. This is because anxiety may elicit symptoms of depression and may also lead to suicidal thoughts [18]. In a meta-analysis, Kanwar et al. [17] examined the relationship between anxiety and suicidal ideation. They found that patients with anxiety were more likely to have suicidal ideation, attempted suicide, and completed suicide compared to those without anxiety [17].

Stress is another risk factor for suicidal ideation, and research has indicated that stress is positively associated with suicidal ideation [19], [20]. In the literature concerning suicide, stress is commonly linked to negative life events and/or negative life experience. There are various work-and-life related stressors, such as stressful life events, loss, unemployment, and other environmental stressors, which could be associated with suicidal ideation [13], [20]. The interactions of various aspects of stressors have the potential to make stress management difficult and have the potential to lead to suicidal ideation [21]. Therefore, stress management requires the integration of a good quality of life, coping skills, and problem solving skills. The way a person copes with stress may indicate how much stress may affect their psychological well-being, how much stress may lead them to feeling a sense of hopelessness or of not being supported, and how they may define stress as a potential contributor to suicidal ideation.

Understanding the psychological factors is important for prevention and treatment in relation to suicide among young people in a specific cultural context. This is because by having a proper understanding about the risk of suicide among adolescents, an appropriate assessment process will facilitate the appropriate treatment being given to those who are at risk. This research has the potential to identify the proper support and psychological needs for those who are having suicidal ideation and at risk of suicide. Therefore, the aims of this study are, firstly, to examine the association between depression, anxiety, stress and suicidal ideation. Secondly, to examine the role of depression, anxiety, and stress in predicting suicidal ideation. In this research, we hypothesize that, firstly, there will be positive correlation between depression, anxiety, stress and suicidal ideation, and, secondly, depression will predict suicidal ideation.

## Method

### Ethical Approval

This study was approved by the Research Ethics Committee, National University of Malaysia (UKM) [Ethics Approval no: NN-064-2013].

### Research Design

The research design was cross-sectional using the survey method. Each participant underwent two psychological tests and a demographic profile.

A total of 190 adolescents, consisting of 103 males and 87 females with an overall mean age of 16.04 (SD = 1.16), participated in this study. The participants were Form 3 to Form 6 students from two schools in Kuala Lumpur. The respondents had obtained parental (including caregivers, and guardians) consent prior to participating in this study. The random sampling method was adopted in this study.

The informed consent of the participants was obtained from parents, caregivers, and guardians on behalf of the minors/children enrolled in this study. As the written informed consent forms were gathered, no verbal consent method was used in this study. The consent of the participants was recorded using informed consent forms distributed to parents, caregivers, and guardians of the participants. The consent procedure and informed consent form were approved by the Research Ethics Committee, National University of Malaysia (UKM).

### Instruments

Two psychological instruments were used in this study – Depression Anxiety Stress Scale 21-item version (DASS-21), and the Beck Scale for Suicide Ideation (BSS).

### Depression Anxiety Stress Scale 21-item version (DASS-21)

DASS-21 is a self-administered psychometric test that gauges the levels of depression, anxiety and stress in the previous week. It is applicable in a clinical or non-clinical setting. There are 21 items in DASS-21 with seven items for each variable measure. The scoring is from 0 to 3 whereby 0 – did not apply to me at all, 1 – applied to me to some degree or some of the time, 2 – applied to me to a considerable degree or a good part of the time, and 3 – applied to me very much or most of the time.

This test takes approximately 10 to 15 minutes to complete. DASS-21 is the most widely validated version of the DASS. Translation to the Malay language was conducted and its psychometric properties were proven [22]. The DASS-21 indicated an alpha coefficient of.74 to.84. The DASS-21 was further validated through factor analysis, which ranged from.39 to.73. The correlation among the scales was between.54 and.68.

### Beck Scale for Suicide Ideation (BSS)

BSS is a 21-item self-administered test to detect and measure the severity of suicidal ideation in adults and adolescents. Both clinical and non-clinical settings are applicable for the usage of BSS. There are three statements under each item in which the intensity ranges from 0 to 2. Participants are asked to circle the statement that best describes their feelings in the past week including the day of administration. The minimum score is 0, while the maximum score is 42. [11].

BSS only takes 10 minutes to complete. It is considered to have good internal consistency at.90 and.87 using Cronbach's Alpha. According to Beck and Steer [23], it is proven that BSS has good content validity with the Suicide Scale of Ideation (SSI) and concurrent validity between SSI and BSS (r = .90).

### Data Analysis

All the raw data were keyed into the SPSS 16.0 program (*Statistical Package for the Social Sciences version 16*) and analysed. Descriptive analysis was mainly used to describe the findings of the data, such as mean, standard deviation and percentage of the demographic data. In addition, inferential analysis was used to test the hypotheses developed. Pearson's correlation was used to test the relationship between the variables. The multiple regression procedure using the ‘Enter step’ was conducted to predict the influence of depression, anxiety and stress on the suicidal ideation of the students.

## Results

Table 1 shows that all the students were aged between 15 and 19 years old (Form 3 to Form 6), with a mean age of 16.04, *SD*  = 1.16. There were 71 students (37.40%) in Form 3, 60 students (31.60%) in Form 4, 43 students in Form 5 (22.60%) and 16 students (8.40%) in Form 6. In terms of the distribution of the students according to religion, there were 14 Muslims (7.40%), 18 Hindus (9.50%), 37 Christians (19.50%) and 113 Buddhists (59.50%) who formed the majority. Most of the participants reported having 2 to 3 siblings, which comprised 32.10% and 37.90%, respectively. Only one student reported having 10 siblings while the others had a number of siblings ranging from 1 to 6. In addition, the findings showed that only 16 students (8.4%) reported having a history of seeking psychological services while 17 students (8.9%) reported that their family members were previously or currently receiving ongoing psychological treatment.

**Table 1:** Demographic characteristics of the sample (N = 190).

Demographic Variable	Frequency	Percentage
**Age**		
Means	16.04	
SD	1.16	
**Level of secondary education/form**		
Form 3	71	37.40
Form 4	60	31.60
Form 5	43	22.60
Form 6	16	8.40
**Religion**		
Islam	14	7.40
Buddhism	113	59.50
Hinduism	18	9.50
Christianity	37	19.50
Others	6	3.20
**Number of siblings**		
1	18	9.50
2	61	32.10
3	72	37.90
4	26	13.70
5	5	2.60
6	4	2.10
10	1	0.50

Table 2 illustrates that the majority of the students scored within the normal range for depression, anxiety and stress, with 46.80%, 40.00% and 57.40%, respectively. There were 24 students (12.60%) who scored mild for depression, while 21 students (11.10%) were in severe depression. The second largest group of students, with 41 students (21.60%), fell in the moderate range of depression, whereas only 8 students (4.20%) fell in the extremely severe depression range. In brief, there was a high percentage of students who experienced moderate to extremely severe depression, although the majority of them (59.40%) were under the normal to mild range. The mean score for the depression scale was 10.98, *SD*  = 9.11.

**Table 2:** Frequency for the levels of depression, anxiety and stress.

Severity	Depression (%)	Anxiety (%)	Stress (%)
Normal	89 (46.80)	76 (40.00)	109 (57.40)
Mild	24 (12.60)	22 (11.60)	27 (14.20)
Moderate	41 (21.60)	40 (21.10)	20 (10.50)
Severe	21 (11.10)	19 (10.00)	18 (9.50)
Extremely Severe	8 (4.20)	23 (12.10)	2 (1.10)

As for the anxiety scale, 22 students (11.60%) reported mild anxiety, while 23 students (12.10%) were found to be in the extremely severe anxiety region. Similar to the depression scale, 40 (21.10%) of the students fell in the moderate range, whereas only 19 students (10.00%) reported experiencing severe anxiety. The findings demonstrated that there was an almost equal distribution of students who were anxious as well as students who were less anxious. The mean score for the anxiety scale was 10.03, *SD*  = 7.73.

The majority of the students reported no or mild stress with 109 students (57.40%) and 27 students (14.20%) for each level. There were 20 students (10.50%) who fell in the moderate range, whereas 18 students (9.50%) reported severe stress. Only two students (1.10%) reported extremely severe stress. Generally, most of the students were under the normal to mild stress level, which signified better stress management. The mean score for the stress scale was 13.88, *SD*  = 7.94.

The results show that 128 students (67.40%) reported no suicidal ideation at all with a score of zero. Approximately 50 students (26.30%) scored 1 to 5 points indicating that, in total, more than two-thirds of the students (93.7%) had no significant suicidal ideation, while 12.6% scored more than 5 points, thereby indicating that they were at risk of suicide.

Table 3 shows that there were positive significant relationships between depression and suicidal ideation; anxiety with suicidal ideation; and stress with suicidal ideation. The results indicated that Pearson's coefficient of depression, anxiety and stress with suicidal ideation were r = .56, r = .42 and r = .47, respectively (*p*<.05). This means that high rates of depression, anxiety and stress result in higher suicidal ideation. These results support the first hypothesis concerning the positive relationship between depression, anxiety, stress and suicidal ideation in the present study.

**Table 3:** Correlation between depression, anxiety, stress and suicidal ideation.

	Suicidal Ideation
Depression	.56*
Anxiety	.42*
Stress	.47*

* *p*<0.05.

The results in table 4 indicate that only one predictor, which was depression, explained a 32.60% of the variance [R^2^ = .33, F(3, 159) = 25.61, *p*<.05]. It was found that only depression significantly predicted suicidal ideation (β = .45, *p*<.05). However, anxiety (β = .08, n.s.) and stress (β = .08, n.s.) were not significant predictors of suicidal ideation. These results support the second hypothesis in that depression is a significant predictor for suicidal ideation in the present study.

**Table 4:** Multiple regression analysis of psychological factors influencing suicidal ideation.

Predictors	B	B	t	p
Constant	−.65		−2.26*	.03*
Depression	.11	.45*	4.36*	.00*
Anxiety	.02	.08	.77	.45
Stress	.02	.08	.73	.47

R^2^ = 0.33.

* *p*<0.05.

## Discussion

The aims of this study are to examine the correlation between depression, anxiety, stress, and suicidal ideation, and to identify role of depression, anxiety, and stress in predicting suicidal ideation.

The findings show that there are moderate positive correlations between depression, anxiety, stress, and suicidal ideation. The results suggest that the respondents who reported high levels of depression tended to report high levels of suicidal ideation. This finding is consistent with other studies concerning the positive association between depression and suicidal ideation, as reported by Yen et al. [15]. In addition, the results also suggest that anxiety is positively associated with suicidal ideation. This suggests that those who reported high levels of stress tended to report high levels of suicidal ideation. This finding is consistent with Kanwar et al. [17]. The correlation analysis also indicates that stress is positively associated with suicidal ideation. This suggests that those who reported high levels of stress tended to report high levels of suicidal ideation. This finding supports other studies concerning the positive association between stress and suicidal ideation [13], [19], [20]. Based on these findings, depression, anxiety, and stress can be considered as the risk factors for suicidal ideation among adolescents in this research.

Although previous studies showed that depression, anxiety and stress were predictors of suicidal ideation [24], [25], in this study, the results indicate that only depression predicts suicidal ideation. This suggests that depression could be regarded as one of the potential predictors for suicidal ideation among adolescents. This means that those who feel depressed may perceive themselves as lacking control over the situation and persistently feeling defeated. In fact, their perception towards their current situation is negative and overwhelming, and they tend to be preoccupied with a sense of guilt and worthlessness. Therefore, depression could be a potential risk for suicidal ideation and suicidal related behaviour among adolescents [26].

However, the findings indicate that anxiety and stress did not predict suicidal ideation. The insignificant predicting role of anxiety could be attributed to the sensitivity of the measure to tap the worry construct. In another study, Carter et al. [24] assessed anxiety, which primarily reflected on worry. For instance, I worry what my parents will say; I worry about what other people think. Worry refers to overwhelming and excessive thoughts about the future, past behaviour and competencies, and peer relationships. Worry is the essential indicator in anxiety disorders and it is present in clinically referred anxious youth that possess suicidal ideation [24]. However, although in the present study DASS-21 was used to measure the anxiety scale, most of the items only reflected anxiety symptoms, such as dryness of mouth, and trembling in the hands. There was only one item that measured the reason for their anxiety. This could partially explain why anxiety could not predict suicidal ideation in the present study. This is because the measurement lacked the indication of worry, which is the key to suicidal ideation in anxious youth. Anxiety symptoms might not be strong indicators of suicidal tendency as they only explain the outcome of worry.

In addition, stress was not a significant predictor of suicidal tendency. This finding contradicted the findings reported by Zhang et al. [27], and Bender et al. [28]. The insignificant predicting role of stress could be partially attributed to the sensitivity of the measure to tap the stress-related construct. Nonetheless, the stress scale in the present study only measures general stress as opposed to adverse life stress as reported by Zhang and colleagues [27]. Hence, the type and intensity of stress, as measured in this study, might not be significant for predicting suicidal ideation. Furthermore, only a handful of studies found that stress directly predicts suicidal ideation. Numerous studies reported that stress and stressful life events were strongly associated with depressive symptoms, which then increased the suicidal risk [25], [29], [30].

Furthermore, it is important to acknowledge the complexity in predicting the role and relationship between depression, anxiety, stress and suicidal ideation. As reported by O'Connor et al. [15], the relationship between depression and suicide ideation is moderated by acute life stress. This means that stress has a vague direct effect on depression, even towards suicidal ideation. This is because stress may precede depression. Therefore, high stress levels could not significantly predict suicidal ideation [15]. In fact, stress is often mediated by other different factors (such as negative appraisal and poor social support) in relation to suicidal ideation. Hence, stress might lead to depression and anxiety but not towards suicidal ideation. Under a stressful environment, suicidal ideation has the potential to increase and individuals will only consider suicidal related behaviour in the presence of the depressive element.

### Limitations and future research

There are a few limitations to the present study. Firstly, as the design of this study is a cross-sectional study, no cause and effect relationship could be established. Secondly, this study did not measure changes in suicide ideation at different time points; therefore, no patterns of change in depression, anxiety, stress, and suicide ideation could be established. Third, the majority of the participants are from urban schools in Malaysia; therefore, the findings could not be generalized to other adolescent populations in Malaysia. Lastly, the current research was limited in terms of its exploration of suicide and suicide related behaviours, as it only focused on suicidal ideation among adolescents.

Given these preliminary findings, future research may focus on longitudinal design and interventional studies in order to explain suicidal ideation more precisely. Other risk and protective factors related to suicidal ideation, such as social support, religiosity, coping and problem solving skills, should be taken into consideration in studying suicidal ideation among adolescents.

### Conclusions

Since prevention is better than cure, identifying risk factors for suicidal ideation is a way to prevent suicide among adolescents. As there are moderate positive correlations between depression, anxiety, stress, and suicidal ideation in the prevention of adolescents' suicide, these risks factors should be assessed and addressed accordingly. As depression predicts suicidal ideation, it is important to screen depression and suicidal ideation among students who are at high risk of suicide. This screening facilitates the prevention of suicide by referring those high-risk students to mental health services. In addition, school counsellors and other mental health professionals play an important role in screening for depression and suicidal ideation among adolescents and offering and/or referring them for the appropriate psychological preventions and interventions.

## References

[1] Smith BL (2014) Psychologists need more training in suicide risk assessment. Monitor on Psychology 45: 42–42.

[2] McIntosh JL, Drapeau CW (for the American Association of Suicidology) (2012) U.S.A. suicide 2010: Official final data. Washington, DC: American Association of Suicidology. Available: http://www.suicidology.org. Accessed 2014 Aug 17.

[3] Barlow DH, Durand VM (2009) Abnormal psychology: An integrative approach (5th ed.). Belmont, CA: Wadsworth Cengage Learning.

[4] Ramachandra S (2010) Suicide rates in Malaysia are on the rise. New Straits Times, 29 August.

[5] Aishvarya S, Maniam T, Hatta Sidi, Oei TPS (2013) Suicide ideation and intent in Malaysia: A review of the literature. Compr Psychiatry 55: 1–6. 23433220 10.1016/j.comppsych.2013.01.005

[6] Institute for Public Health. (2011) National Health and Morbidity Survey 2011. Kuala Lumpur: Ministry of Health Malaysia.

[7] Lum M (2011) I'm gonna jump. *The Star Online*, 20 November. Available: http://thestar.com.my/health/story.asp?file=/2011/11/20/health/9917622&sechealth. Accessed 17 August 2014.

[8] Tam CL, Lee TH, Har WM, Chan LC (2011) Perception of suicidal attempts among college students in Malaysia. Asian Soc Sci 7: 30–41.

[9] Gutierrez PM, Osman A (2008) Adolescent suicide: an integrated approach to the assessment of risk and protective factors. DeKalb, IL: Northern Illinois University Press.

[10] Gutierrez PM, Osman A, Kopper BA, Barrios FX (2000) Why young people do not kill themselves: the Reasons for Living Inventory for Adolescents. J Clin Child Psychol 29: 177–187. 10802827 10.1207/S15374424jccp2902_4

[11] Phillips MR, Li XY, Zhang YP (2002) Suicide rates in China 1995–99. Lancet 359: 835–40. 11897283 10.1016/S0140-6736(02)07954-0

[12] Shemesh E, Annunziato RA, Rubinstein D, Sultan S, Malhotra J, et al (2009) Screening for depression and suicidality in patients with cardiovascular illnesses. Am J Cardiol 104: 1194–1197. 19840561 10.1016/j.amjcard.2009.06.033

[13] Rohtash S, Hardeep LJ (2008) Suicidal Ideation in Relation to Depression, Life Stress and Personality among College Students. J Indian Acad Appl Psychol 34: 259–265.

[14] Takeuchi T, Nakao M (2013) The relationship between suicidal ideation and symptoms of depression in Japanese workers: a cross-sectional study. BMJ Open 3: e003643 10.1136/bmjopen-2013-003643 PMC3845061 24293204

[15] Yen S, Shea MT, Pagno M, Sanislow CA, Grilo CM (2003) Axis I and axis II disorders as predictors of prospective suicide attempts: findings from the collaborative longitudinal personality disorders study. J Abnorm Psychol 112: 375–381. 12943016 10.1037/0021-843x.112.3.375 PMC3272767

[16] McDowell AK, Lineberry TW, Bostwick JM (2011) Practical Suicide-Risk Management for the Busy Primary Care Physician. Mayo Clin Proc 86: 792–800 10.4065/mcp.2011.0076 21709131 PMC3146379

[17] Malik S, Kanwar A, Sim LA, Prokop LJ, Wang Z, et al (2014) The association between sleep disturbances and suicidal behaviors in patients with psychiatric diagnoses: a systematic review and meta-analysis. Syst Rev 3: 18 10.1186/2046-4053-3-18 24568642 PMC3945796

[18] Davidson CL, Wingate LR, Grant DM, Judah MR, Mills AC (2011) Interpersonal Suicide Risk and Ideation: The Influence of Depression and Social Anxiety. J Soc Clin Psychol 30: 842–855.

[19] Gould MS, Greenberg T, Velting D, Shaffer D (2003) Youth suicide risk and preventive interventions: a review of the past 10 years. J Am Acad Child Adolesc Psychiatry 42: 386–405. 12649626 10.1097/01.CHI.0000046821.95464.CF

[20] Overholser JC (2003) Predisposing factors in suicide attempts: life stressors. In ASpirito & JOverholser (Eds), Evaluating and Treating Adolescent Suicide Attempters: From Research to Practice (pp. 42–49). New York: Academic Press.

[21] Lee SI, Jung H (2006) Psychosocial Risk Factors for Suicide. Psychiatr Invest 3: 15–22.

[22] Ramli M, Mohd AF, Zaini Z (2007) Translation, validation andpsychometric properties of Bahasa Malaysia version of the DepressionAnxiety and Stress Scale (DASS). ASEAN J Psychiatr 8: 82–89.

[23] Beck AT, Steer RA (1993) Beck Scale for Suicide Ideation: Manual. San Antonio, TX: The Psychological Corporation.

[24] Carter R, Silverman WK, Allen A, Ham L (2008) Measures matter: The relative contribution of anxiety and depression to suicidal ideation in clinically referred anxious youth using brief versus full length questionnaires. Depress Anxiety 25: E27–E35. 10.1002/da.20468 18729149

[25] O'Connor RC, Rasmussen S, Hawton K (2010) Predicting depression, anxiety and self-harm in adolescents: The role of perfectionism and acute life stress. Behav Res Ther 48: 52–59. 19818954 10.1016/j.brat.2009.09.008

[26] Lai YC, Shek TL (2010) Personal and family correlates of suicidal ideation in Chinese adolescents in Hong Kong. Soc Indic Res 95: 407–419.

[27] Zhang XY, Wang HP, Xia Y, Liu XH, Jung EJ (2011) Stress, coping and suicide ideation in Chinese college students. J Adolesc 35: 1–8. 22032975 10.1016/j.adolescence.2011.10.003

[28] Bender WN, Rosenkrans CB, Crane MK (1999) Stress, depression and suicideamong students with learning disabilities: Assessing the risk. Learn Disabil Q 22: 143–156.

[29] Hernandez BN (2006) Psychosocial predictors of depressive symptoms in Mexican American middle school girls. Thesis Ph.D. University of Houston.

[30] You Z, Chen M, Yang S, Zhou Z, Qin P (2014) Childhood Adversity, Recent Life Stressors and Suicidal Behavior in Chinese College Students. PLoS ONE 9(3): e86672 10.1371/journal.pone.0086672 24681891 PMC3969373

